# Noninvasive imaging of hollow structures and gas movement revealed the gas partial‐pressure‐gradient‐driven long‐distance gas movement in the aerenchyma along the leaf blade to submerged organs in rice

**DOI:** 10.1111/nph.17726

**Published:** 2021-09-30

**Authors:** Yong‐Gen Yin, Yoshinao Mori, Nobuo Suzui, Keisuke Kurita, Mitsutaka Yamaguchi, Yuta Miyoshi, Yuto Nagao, Motoyuki Ashikari, Keisuke Nagai, Naoki Kawachi

**Affiliations:** ^1^ Takasaki Advanced Radiation Research Institute National Institutes for Quantum and Radiological Science and Technology (QST) Gunma 370‐1292 Japan; ^2^ Bioscience and Biotechnology Center Nagoya University Aichi 464‐8601 Japan; ^3^ Materials Sciences Research Center Japan Atomic Energy Agency (JAEA) Ibaraki 319‐1195 Japan

**Keywords:** ^11^CO_2_ tracer, [^13^N]N_2_ tracer, aeration, deepwater rice, gas movement, hollow structure, PETIS imaging, X‐ray CT

## Abstract

- Rice (*Oryza sativa*) plants have porous or hollow organs consisting of aerenchyma, which is presumed to function as a low‐resistance diffusion pathway for air to travel from the foliage above the water to submerged organs. However, gas movement in rice plants has yet to be visualized in real time.
- In this study involving partially submerged rice plants, the leaves emerging from the water were fed nitrogen‐13‐labeled nitrogen ([^13^N]N_2_) tracer gas, and the gas movement downward along the leaf blade, leaf sheath, and internode over time was monitored.
- The [^13^N]N_2_ gas arrived at the bottom of the plant within 10 min, which was 20 min earlier than carbon‐11 photoassimilates. The [^13^N]N_2_ gas movement was presumably mediated by diffusion along the aerenchyma network from the leaf blade to the root via nodes functioning as junctions, which were detected by X‐ray computed tomography.
- These findings imply the diffusion of gas along the aerenchyma, which does not consume energy, has enabled plants to adapt to aquatic environments. Additionally, there were no major differences in [^13^N]N_2_ gas movement between paddy rice and deepwater rice plants, indicative of a common aeration mechanism in the two varieties, despite the difference in their response to flooding.

## Introduction

Organisms living in diverse habitats face a wide range of abiotic stresses. Plants in aquatic environments have advantages regarding water uptake, but they are at risk of oxygen (O_2_) and CO_2_ deficiency when submerged because of flooding. The dissolved gas diffusion rates are *c*. 10^4^‐fold lower in water than in air. Therefore, the O_2_ content of submerged plant tissues can be low, which restricts respiration (Armstrong, 1980). Unless sufficient O_2_ is supplied from the aerial organs, the submerged organs of terrestrial plants can become deficient in O_2_, leading to restricted respiration and inhibited plant growth. A limited CO_2_ supply can also restrict the photosynthesis in the submerged leaves of terrestrial plants (Mommer & Visser, 2005; Colmer & Pedersen, 2008a). Thus, the aeration of submerged organs is essential for terrestrial plants in flood‐prone environments.

Rice (*Oryza sativa*) is a staple crop that is cultivated in waterlogged soil, with its roots and basal shoot parts submerged during most of its life cycle. Rice possesses various traits that allow it to grow in paddy fields. Of particular importance is the aeration of the submerged plant parts through porous or hollow organs consisting of aerenchyma, which forms along the leaf blade (LB), leaf sheath (LS), internode (IN), and root (Raskin & Kende, 1984; Colmer & Pedersen, 2008b). These air‐filled spaces are presumed to function as a low‐resistance diffusion pathway for the transfer of air from the leaves emerging from the water to submerged organs. Hence, the O_2_ is retained in the submerged organs of rice plants (Thomson *et al*., 1990; Pedersen *et al*., 2009; Winkel *et al*., 2013). Deepwater rice adapts to flooding by rapidly elongating its INs to allow the leaves remaining above the water surface to match the height of rising floodwaters. This trait enables rice plants to remain in contact with the air under flooding conditions, thereby maintaining respiration through the leaves that emerge from the water. The air layer formed on submerged leaves (leaf gas film) is important for the tolerance to submergence. Specifically, it enlarges the area of the interface between the air and water to enable air to enter submerged organs, thereby promoting the movement of gas to the underwater plant parts (Raskin & Kende, 1983, 1985; Winkel *et al*., 2014). A recent study revealed that the ‘snorkeling’ of air from the emerged shoot tops to the submerged organs is important for the maintenance of the O_2_ content in the pith cavity of partially submerged paddy rice and deepwater rice (Mori *et al*., 2019). The importance of aeration for paddy rice and deepwater rice has been discussed from physiological and anatomical perspectives. However, because of technical difficulties, there is no direct evidence for the long‐distance gas movement through the aerenchyma from rice organs above water to those underwater. Moreover, the underlying mechanism remains uncharacterized.

A positron‐emitting tracer imaging system (PETIS) is a noninvasive live imaging system that detects and visualizes the gamma rays emitted from a positron‐emitting radionuclide (e.g. cadmium‐107 (^107^Cd), carbon‐11 (^11^C), nitrogen‐13 (^13^N), zinc‐65, or sodium‐22) that is taken in by a test plant placed between a pair of PETIS detector heads (Uchida *et al*., 2004; Fujimaki *et al*., 2010). This system has been used to observe and quantify radiotracer movements in intact living plants. For example, Fujimaki *et al*. (2010) used a PETIS to investigate the absorption, transport, and accumulation of Cd in living rice plants after the roots were fed ^107^Cd. Additionally, C fixation and photoassimilate transport via the phloem to sink organs in soybean, tomato, and strawberry plants were visualized noninvasively after the leaves were fed a ^11^CO_2_ tracer (Kawachi *et al*., 2011; Yamazaki *et al*., 2015; Hidaka *et al*., 2019). N fixation by *Rhizobium leguminosarum* in soybean has been observed using [^13^N]N_2_ gas as a tracer produced by a high‐purity and high‐yield purification system for a PETIS analysis (Yin *et al*., 2019). These studies, as well as ongoing research, have confirmed the utility of the PETIS for dissecting physiological phenomena inside living plants, making this system a powerful tool for elucidating the mechanisms underlying long‐distance gas movement.

The objectives of this study were to visualize and evaluate the gas movement from the leaves in contact with the air to the submerged organs in partially submerged living rice plants to clarify the aeration mechanisms under flooding conditions. The [^13^N]N_2_ tracer gas was used to visualize the gas movement through the aerenchyma in intact paddy rice T65 and deepwater rice C9285 plants or in artificial tubes using a PETIS. The movement of [^13^N]N_2_ gas along the aerenchyma was markedly faster than the movement of ^11^C‐labeled photoassimilates derived from ^11^CO_2_ photosynthesis via the phloem, reflecting the benefit of transporting substrates in a gaseous state. Moreover, the movements of [^13^N]N_2_ gas in T65 and C9285 rice plants were quantitatively compared.

## Materials and Methods

### Plant materials and growth conditions

A deepwater rice variety (*Oryza sativa* L. var. C9285 syn. Dowai38/9) and a paddy rice variety (*O. sativa* var. Taichung 65) were used in PETIS experiments to visualize the long‐distance gas movement in the plant body. Seeds were sterilized in hot water (60°C) for 10 min and then stored at 4°C for 24 h. To induce germination, the seeds were imbibed in deionized water at 30°C for 72 h. Germinated seeds were sown in pods (9.0 × 9.0 × 13.5 cm^3^) filled with soil suitable for rice (N, P, and K contents of 0.25 g kg^−1^ soil, 0.3 g kg^−1^ soil, and 0.25 g kg^−1^ soil, respectively; Mikawabaido; Aichi Mederu Co. Ltd, Aichi, Japan). Plants were grown for 45 d after germination in a glasshouse at about 26°C and under natural sunlight.

A normal Japanese cultivar (*O. sativa* L. cv. Nipponbare) was used in X‐ray computed tomography (CT) experiments to visualize the internal hollow structures of rice plants. Seeds were sterilized with sodium hypochlorite and germinated in deionized water for 1 wk. The seedlings were transferred to a container filled with culture solution comprising 0.17 mM Na_2_HPO_4_, 0.70 mM (NH_4_)_2_SO_4_, 0.27 mM K_2_SO_4_, 0.47 mM MgSO_4_, 0.37 mM CaCl_2_, 0.045 mM FeC_6_H_5_O_e_·nH_2_O (Fe citrate), 0.16 μM CuSO_4_, 0.15 μM ZnSO_4_, 0.10 μM Na_2_MoO_4_, 15 μM H_3_BO_3_, 4.6 μM MnSO_4_, and 0.34 mM SiO_2_. The culture solution was renewed weekly, and its pH was adjusted to 5.5 daily. The seedlings were grown in a growth chamber at 30°C with a 12 h : 12 h, light : dark, cycle. At approximately 8 wk after sowing (1 wk after the emergence of the first ear), a test plant was selected for the X‐ray CT experiment.

### Production of [^13^N]N_2_ and ^11^CO_2_ tracer gases

N_2_, which is an inert gas, was used to exclude plant effects and evaluate the gas movement in the hollow structures of rice plants. A positron‐emitting radionuclide, ^13^N, was generated via the ^16^O(p,α)^13^N nuclear reaction induced by the irradiation of pure CO_2_ target gas using 18.3 MeV protons. An azimuthally varying field cyclotron located at Takasaki Ion Accelerators for Advanced Radiation Application (TIARA), National Institutes for Quantum and Radiological Science and Technology (Gunma, Japan) was used to deliver the protons. The irradiated gas containing [^13^N]N_2_, [^13^N] nitrogen oxides (NO*_x_*), and CO_2_ was used immediately to purify [^13^N]N_2_ as described by Yin *et al*. (2019). The CO_2_ was removed using soda lime and the [^13^N]NO*_x_* was deoxidized to [^13^N]N_2_ using reduced copper at 600°C. The resulting [^13^N]N_2_ was mixed with ambient air in a gas‐tight syringe to prepare [^13^N]N_2_ tracer gas. Additionally, several [^13^N]N_2_ tracer gases with varying N_2_ compositions (100%, 50%, and 20%) were prepared by mixing with helium gas in a gas‐tight syringe.

To evaluate the phloem transport of photosynthetic C and compare it with the gas movement in rice plants, leaves were fed the ^11^CO_2_ tracer. The ^11^C was produced by the ^14^N(p,α)^11^C reaction, which was catalyzed by bombarding pure N_2_ gas with 10 MeV protons from the azimuthally varying field cyclotron at TIARA (Yamazaki *et al*., 2015). After the irradiation, the resulting gas contained a small amount of ^11^CO_2_ and a large amount of the target gas (N_2_). The ^11^CO_2_ gas was collected as dry ice by passing the irradiated gas through a stainless steel pipe cooled with liquid N_2_. The ^11^CO_2_ tracer was extracted from the stainless steel pipe using ambient air at room temperature and collected in a gas‐tight syringe.

### Positron‐emitting tracer imaging system experiment

The PETIS apparatus was modified from a PPIS‐4800 system (Hamamatsu Photonics K.K., Hamamatsu, Japan) and installed in a growth chamber for plant studies. It had two opposing detector heads to detect the annihilation gamma rays from the positron‐emitting nuclides (e.g. ^13^N and ^11^C) in real time and continuously construct a two‐dimensional image in the middle plane (Uchida *et al*., 2004; Kawachi *et al*., 2011).

**Fig. 1:**
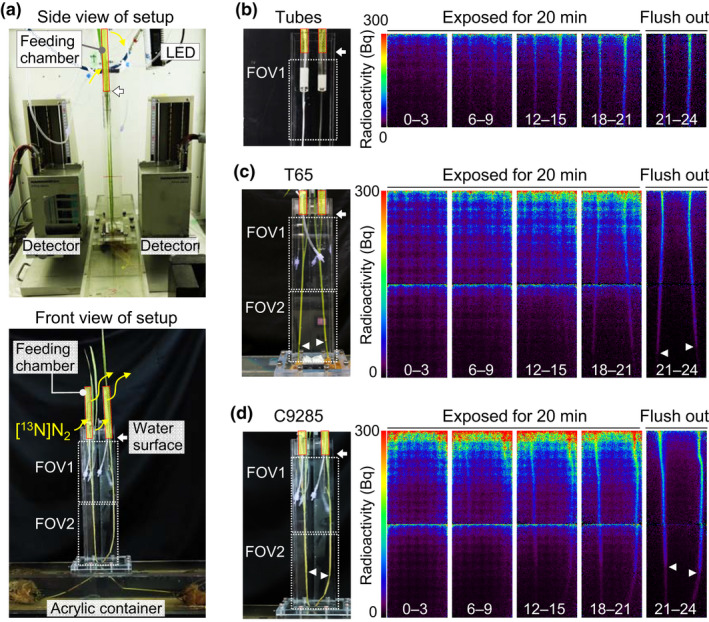
Experimental setup for analyzing nitrogen‐13‐labeled nitrogen ([^13^N]N_2_) gas movement in paddy rice T65 and deepwater rice C9285. (a) Experimental setup for monitoring the [^13^N]N_2_ tracer gas. The T65 and C9285 plants were placed in a water tank and then partially submerged. Artificial tubes with 0.8 mm (left) and 3.18 mm (right) diameters were treated similarly as controls. A mature leaf was fed the [^13^N]N_2_ tracer gas in a feeding chamber. After a 20 min feeding, the tracer gas was flushed out. The upper and lower areas were established as the fields of view (FOV1 and FOV2, respectively). The FOV was 12 × 19 cm (width and height, respectively). Two replicate measurements on each of two individuals T65 and C9285 plants were examined. (b–d) Direct observations of [^13^N]N_2_ tracer gas movement in (b) artificial tubes 0.8 and 3.18 mm in diameter and in (c) T65 and (d) C9285 rice plants. The ^13^N radioactivity is indicated by color. The mean radioactivity is presented at 3 min intervals. The numbers in white on each image indicate the time after gas feeding. Gas movements were observed in artificial tubes and in the T65 and C9285 plants. White arrowheads indicate the node position in each rice plant.

To observe the gas movement in the hollow structures of plants, we constructed a system for visualizing the movement of the inert [^13^N]N_2_ tracer gas. Two artificial tubes (0.8 and 3.18 mm diameter) were used as controls for analyses of gas movement in paddy rice T65 and deepwater rice C9285 plants. The test plant or artificial tube was positioned in an acrylic container and then submerged in tap water, with only a part of the tube or the upper LBs exposed above the water surface and in contact with the atmosphere (Supporting Information Fig. S1). The acrylic container with the test sample was subsequently placed at the midpoint between the opposing detector heads of the PETIS (Fig. 1a). The growth chamber where the PETIS is installed was maintained at 26°C with 65% relative humidity, and the light level was maintained at 1000 µmol m^−2^ h^−1^ at the top of the plants throughout the imaging experiments. The youngest fully expanded leaf or artificial tube above the water surface was covered with a gas‐tight acrylic feeding chamber containing a gas inlet and outlet connection tubes. The feeding chamber width, depth, and height were 12 mm, 12 mm, and 300 mm, respectively, and part of it was submerged in the water to block the release of the [^13^N]N_2_ or ^11^CO_2_ tracer gas to the atmosphere.

A gas‐tight syringe containing 25 ml [^13^N]N_2_ tracer gas (60–160 MBq ^13^N radioactivity) was connected to the inlet of the feeding chamber to feed each leaf or artificial tube with the tracer gas, after which the PETIS experiment was initiated immediately for 60 min. After 20 min of tracer feeding, the tracer gas was forced out at a rate of 100 ml min^−1^ by a pump connected to the inlet of the feeding chamber. The field of view (FOV) of the PETIS detector heads (12 cm and 19 cm in width and height, respectively) was set on the upper area (FOV1) of the artificial tube (Fig. 1b) and on the upper (FOV1) or lower (FOV2) area of the submerged rice plants (Fig. 1c,d; Video S1). A gas‐tight syringe was used to feed the artificial tube with different [^13^N]N_2_ tracer gases (100%, 50% and 20% N_2_), after which the PETIS experiment was initiated immediately (Fig. S2). The PETIS experiment was repeated using the same tubes or plants after the ^13^N signal decayed sufficiently for each FOV.

After the ^13^N radioactivity had decayed sufficiently, the ^11^CO_2_ feeding experiment was conducted using a feeding method similar to that for [^13^N]N_2_ gas. To observe ^11^C movement and compare with the [^13^N]N_2_ gas movement, the same leaf was fed 25 ml ^11^CO_2_ tracer gas (30 MBq ^11^C radioactivity) collected in a gas‐tight syringe as a pulse using the same protocol as that used for the [^13^N]N_2_ tracer gas feeding. The PETIS experiment was then initiated immediately for 120 min. The FOV was set on the lower area (FOV2) of the submerged T65 or C9285 plants (Video S1, right).

All of the PETIS images of the ^13^N or ^11^C distribution in the rice plants or artificial tubes were corrected for signal decay on the basis of the half‐life of ^13^N (9.97 min) or ^11^C (20.39 min), respectively. The PETIS images provided quantitative information regarding the distribution of ^13^N or ^11^C radioactivity (becquerels) because the PETIS detector was corrected according to the sensitivity distribution and the counting efficiency of the gamma rays emitted from a standard positron‐emitting radiation source.

### Analysis of positron‐emitting tracer imaging system image data

Serial image data for the [^13^N]N_2_ gas or the ^11^C‐photoassimilates movement from the LB to the IN via the LS were generated by integrating the original PETIS image data acquired from the gamma‐rays measurement data as counts at 10 s per frame. The counts of every 18 frames of the image data were averaged and integrated to 180 s per frames. All image data were corrected to amount of the radioactivity at 3 min intervals for analyzing [^13^N]N_2_ gas or ^11^C‐photoassimilate distribution using ImageJ (v.1.51J8) software (https://imagej.nih.gov/ij/). All [^13^N]N_2_ gas movement image data for the test plants and artificial tubes were normalized against the same radioactivity feeding levels. Additionally, two FOVs per plant were combined to produce one image based on the normalization (Fig. 1c,d; Video S1).

**Fig. 2:**
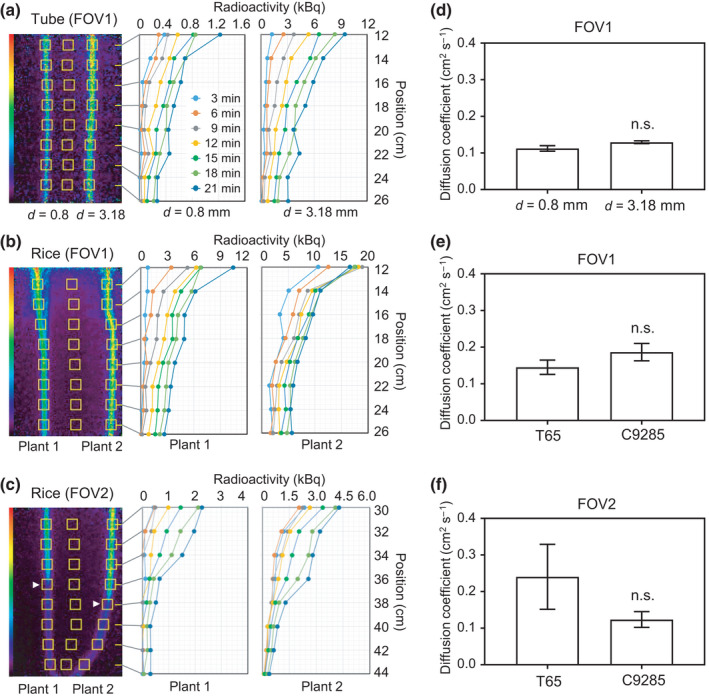
Quantification of nitrogen‐13 (^13^N) radioactivity and calculation of the diffusion coefficients for the artificial tubes and rice plants. (a–c) Several regions of interest (ROIs; yellow square boxes) were set for the positron‐emitting tracer imaging system images. The changes in ^13^N radioactivity in sequential ROI positions at 3 min intervals are presented as different colored lines. The ^13^N signal intensity was calculated using the data for the (a) artificial tubes or the (b) and (c) C9285 plants presented in Fig. 1(b) or (c,d), respectively. Two repeated measurements on two individual plants (*n* = 4) were analyzed. Colored broken lines indicate the changes in radioactivity in each ROI at the same time point. The positions of each ROI are indicated as distances from the bottom of the feeding chamber. The quantitative analysis revealed similar trends for T65 (data not shown). (d–f) Diffusion coefficients for the (d) artificial tubes and the rice plants in (e) field of view (FOV)1 or (f) FOV2. No significant (n.s.) differences were detected by the one‐way ANOVA with significance level of 0.05. Error bars indicate SE. *n* = 4.

Eight 11 × 11 mm squares were defined as regions of interest (ROIs) in each rice plant or tube. The same square set in between two ROIs was defined as the background (Fig. 2a–c, left). After subtracting the background, the ^13^N radioactivity of each ROI of the rice plant or tube was used to generate a curve for the radioactivity distribution changes at increasing distances from the feeding chamber at 3 min intervals (Fig. 2a–c, right). The [^13^N]N_2_ gas diffusion coefficient for each artificial tube or test plant was calculated using the following equations (Figs 2d–f, 4b, S2). We assumed that the N concentration ϕ(x,t) (^13^N radioactivity) inside the tube or rice plant at time *t* and position *x* satisfied the one‐dimensional diffusion equation of gas (Eqn 1). Eqn 2 was obtained by assuming the initial condition at *t* = 0 was ϕ(x,0)=0 and the boundary condition at *x* = 0 was ϕ(0,t)=constant:

(Eqn 1) $$\frac{\partial \phi }{\partial t}=D\frac{\partial^{2}\phi }{\partial x^{2}}$$

(Eqn 2) $$\phi \left(x,t\right)=\phi_{0}\left(1‐\frac{2}{\sqrt{\pi }}\int_{0}^{\frac{x}{\sqrt{4Dt}}}e^{‐\sigma^{2}}\;d\sigma \right)$$

(*D*, the diffusion coefficient). The diffusion coefficient was obtained by fitting the experimental data with Eqn 2. Both *ϕ*_0_ and *D* were used as fitting parameters. A typical movie of the [^13^N]N_2_ gas movement, which is the source of the aforementioned analysis, is provided in Video S1.

**Fig. 3:**
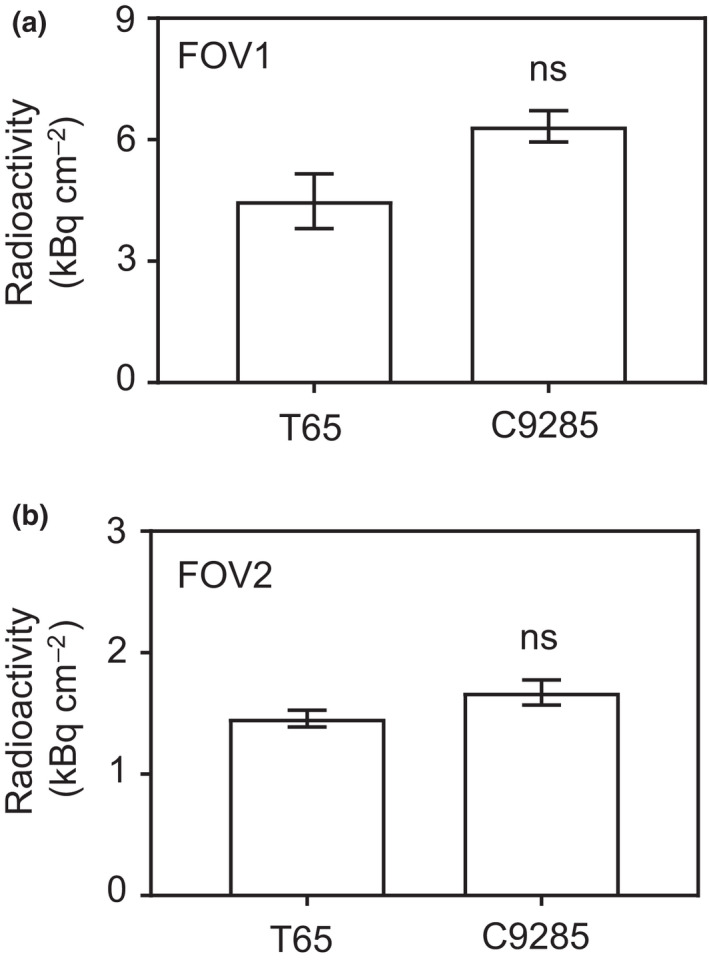
Total amounts of nitrogen‐13‐labeled nitrogen ([^13^N]N_2_) gas after 20 min (field of view (FOV)1) or 40 min (FOV2) in T65 and C9285 plants. Mean ^13^N radioactivities in T65 and C9285 plants were calculated at (a) 20 min after feeding for FOV1 or at (b) 40 min after feeding for FOV2 in Fig. 1(c) and (d), respectively. No significant (ns) differences were detected between T65 and C9285 plants in FOV1 or FOV2 by the one‐way ANOVA with significance level of 0.05. *n* = 4. Error bars indicate SE.

**Fig. 4:**
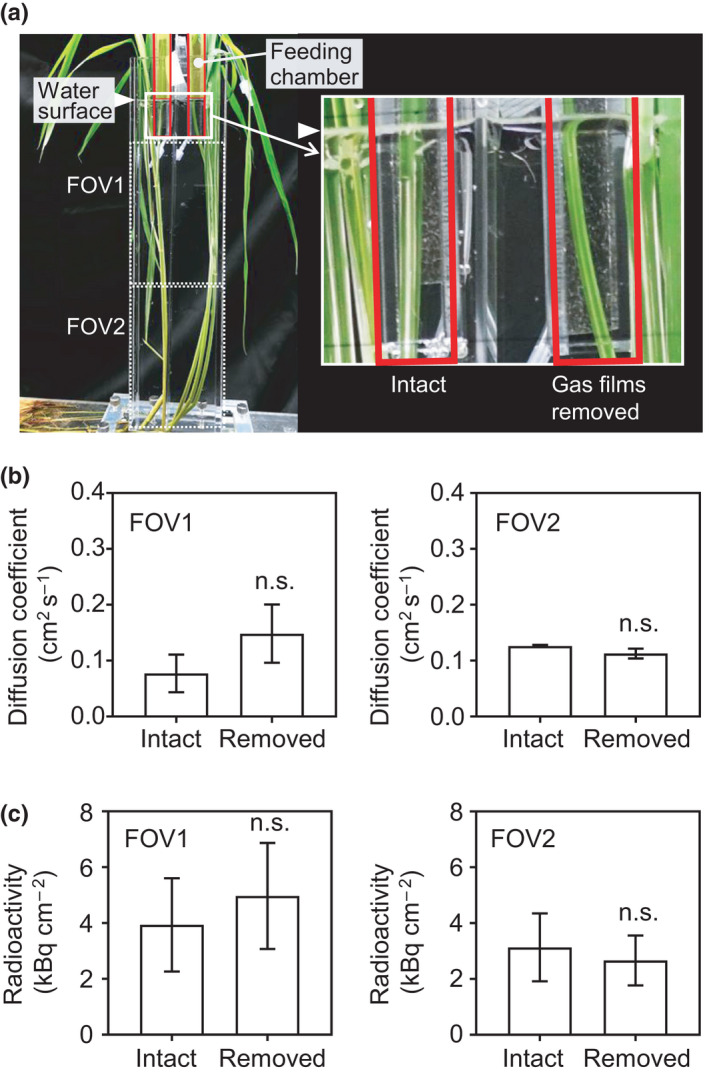
Effects of gas film removal on of nitrogen‐13‐labeled nitrogen ([^13^N]N_2_) gas movement in C9285 plants. (a) Removal of the gas film from a leaf, which was confirmed by the lack of a reflection of light on the area treated with Triton X‐100. White arrowheads indicate water surface. (b) Diffusion coefficients calculated using the data for field of view (FOV)1 and FOV2. (c) Mean ^13^N radioactivities for C9285 plants in FOV1 and FOV2. Open circles indicate replicates. No significant (ns) differences were detected by the one‐way ANOVA with significance level of 0.05. *n* = 3. Error bars indicate SE.

**Fig. 5:**
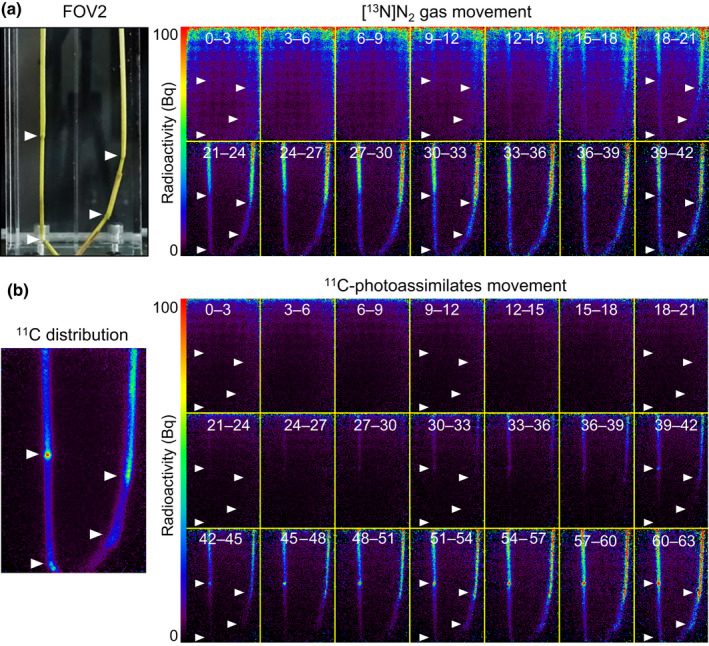
Comparison between the nitrogen‐13‐labeled nitrogen ([^13^N]N_2_) gas and carbon‐11 (^11^C)‐photoassimilate movements. (a) The [^13^N]N_2_ gas movement in field of view 2 (FOV2) in Fig. 1(d) is presented for comparison. (b) ^11^C‐photoassimilate movement. The C9285 plant used in the [^13^N]N_2_ gas tracer experiment (Fig. 1d) was fed ^11^CO_2_ tracer gas after the ^13^N signal decayed. White arrowheads indicate nodes.

**Fig. 6:**
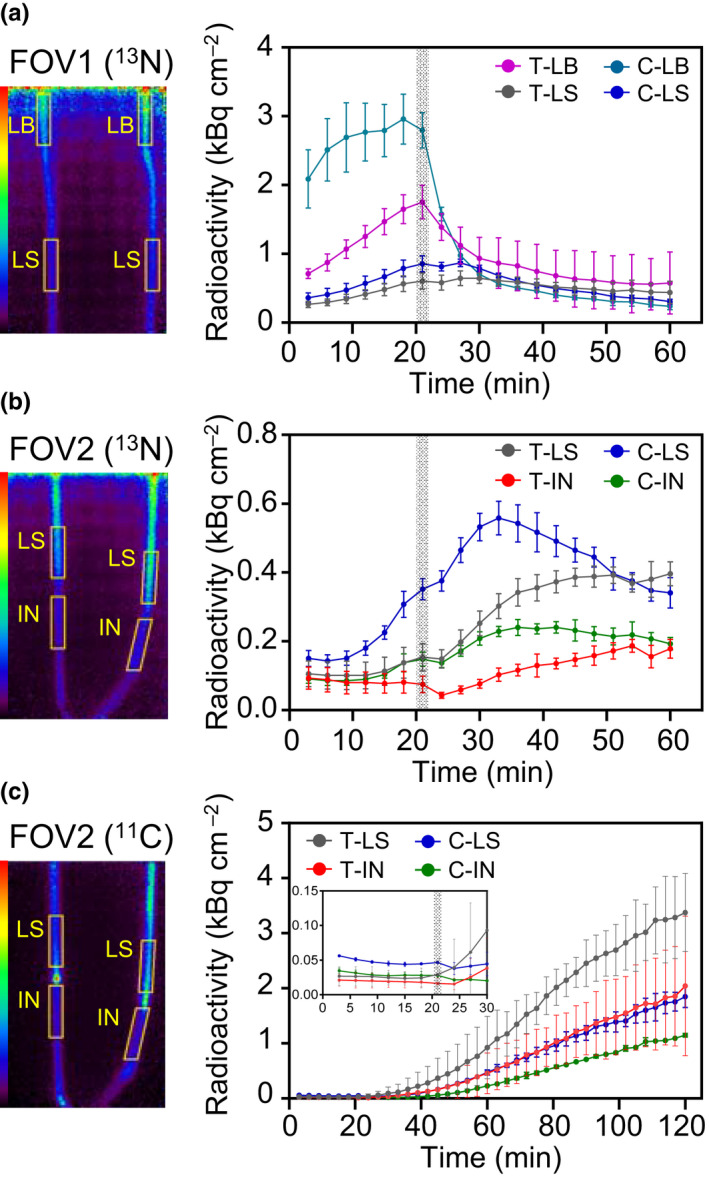
Quantification of the radioactivity during time–activity curves for the analysis of nitrogen‐13‐labeled nitrogen ([^13^N]N_2_) gas and carbon‐11 (^11^C)‐photoassimilate movement in T65 and C9285 plants. The positions of the regions of interest set on (a) field of view (FOV)1 or (b, c) FOV2 in T65 (T‐LB, leaf blade; T‐LS, leaf sheath; T‐IN, internode) and C9285 (C‐LB, leaf blade; C‐LS, leaf sheath; C‐IN, internode). (a) [^13^N]N_2_ gas movement in FOV1. (b) [^13^N]N_2_ gas movement in FOV2. (c) ^11^C‐photoassimilate movement in FOV2. Four replicates of T65 and C9285 plants were examined in (a, b) and two replicates of T65 and C9285 plants in (c). Error bars indicate SD.

We calculated the ^13^N radioactivity levels of the [^13^N]N_2_ gas arriving at FOV1 within 20 min and at FOV2 within 40 min of each test plant being fed the tracer (Figs 3, 4c). Time–activity curves (TACs) were generated on the basis of the radioactivity changes over time (Fig. 5; Video S1) for each ROI on the LB, LS, or IN located at FOV1 and FOV2, as indicated in the PETIS images in Fig. 6 (left). The radioactivity data were normalized against the leaf area (square centimeters) covered by the feeding chamber (Fig. 6).

### Image acquisition and analysis of the hollow structures

**Fig. 7:**
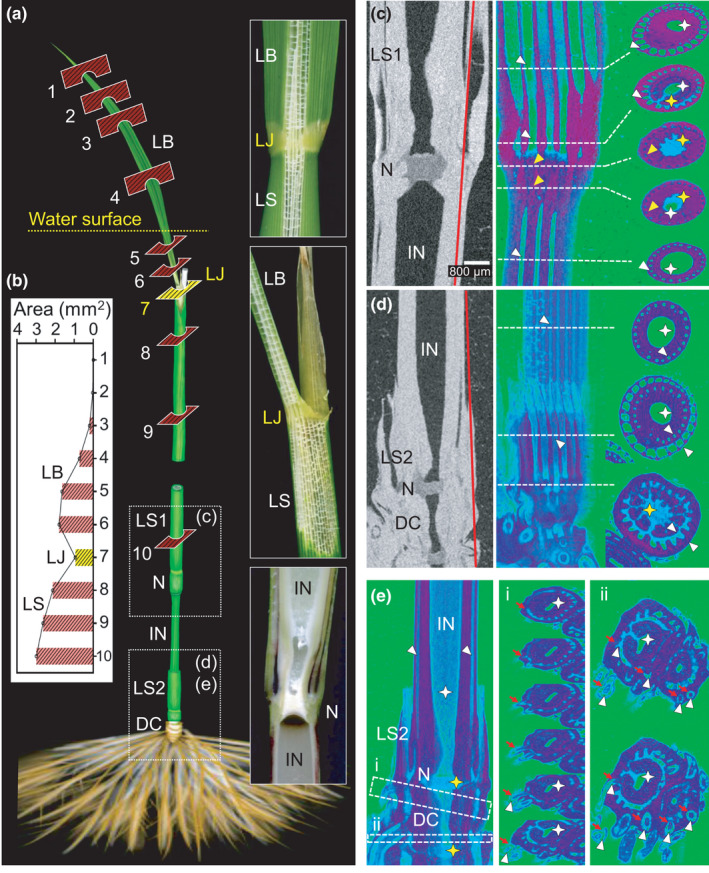
Plant body hollow structures. (a) Anatomy of the hollow structures in the leaf blade (LB), the leaf sheath (LS) including the lamina joint (LJ), and the internode (IN) with a node (N). (b) Area of the aerenchyma in each position indicated in (a). (c–e) Acquisition of two‐dimensional segmentation data for the X‐ray computed tomography (CT) image of a rice plant. The right panel corresponds to the two‐dimensional longitudinal image along the red line in the left panel. The hollow structure (empty or air) is in green, the parenchyma is in red, and the spongy tissue is in blue. White arrowhead, hollow structure in LS and IN; yellow arrowhead, aerenchyma with spongy tissue in the node; white star, hollow structure in IN; yellow star, spongy tissue in the node; red arrows, roots; DC, discrimination center. The scale bar in (c) applies to all X‐ray CT images (c–e).

The internal hollow structures of the rice plants were visualized using a digital camera after removing the epidermal tissue or part of the rice plant using a razor blade (Fig. 7a, right). The hollow structure area was determined from the tissue sections prepared from representative sample positions, as shown in Fig. 7(a) (1–10), using ImageJ software (Fig. 7b).

The connection between the aerenchyma along the leaf sheath and the root via the node and IN was noninvasively visualized by X‐ray CT (Fig. 7c–e). The test plant (*c*. 10 cm above the root base) was analyzed using an XT H 225 (Nikon Solutions Co. Ltd, Tokyo, Japan) three‐dimensional X‐ray CT system. The test plant was scanned at a constant voltage of 95 kV and a current of 185 μA. A total of 2172 images were used to reconstruct a three‐dimensional image using the X‐ray CT system software. The image data obtained were used to determine the morphological traits of the hollow structures in the LS, node, IN, discrimination center, and root.

## Results

### [^13^N]N_2_ gas movement in paddy rice T65 and deepwater rice C9285 plants

The long‐distance movement of [^13^N]N_2_ tracer gas was continuously visualized for 60 min in artificial tubes and rice plants (Fig. 1). Within 9 min of exposure, the [^13^N]N_2_ gas was visualized as a linear shape along the artificial tubes (Fig. 1b). There were no particular differences in the movement trends between the two artificial tubes with 0.8 mm (left) and 3.18 mm (right) diameters. The [^13^N]N_2_ gas reached the bottom of FOV1 within 15 min of gas feeding. Similarly, a downward [^13^N]N_2_ gas movement was observed in the partially submerged T65 and C9285 rice plants (Fig. 1c,d). The ^13^N signal intensity gradually increased over time along the LB and LS toward the IN. The ^13^N signal intensity continued to increase until the gas was flushed out at 20 min. The [^13^N]N_2_ tracer gas moved to the bottom of FOV2, which was more than 40 cm away from the feeding chamber (Fig. 1c,d). An examination of the [^13^N]N_2_ distribution after the gas was flushed out (i.e. 21–24 min after gas feeding) revealed that the ^13^N signals gradually decreased from the LS (FOV1) to the IN (bottom of FOV2) in both the T65 and C9285 plants. Notably, ^13^N signals decreased from the node position (indicated the white arrowheads in Fig. 1c,d, right). These results reflected the long‐distance downward [^13^N]N_2_ gas movement in artificial tubes and rice plants. Additionally, [^13^N]N_2_ gas moved down to the submerged organs in T65 and C9285 plants in a manner similar to that in the artificial tubes.

### Quantitative analysis of [^13^N]N_2_ gas movement

The ^13^N signal intensity at each ROI (indicated by yellow square boxes in Fig. 2a–c, left) was quantitatively analyzed at 3 min intervals for the artificial tubes and the T65 and C9285 plants. The higher‐to‐lower ^13^N radioactivity intensity gradient from the upper‐to‐lower ROIs was detected in the same timeline for the artificial tubes with differing diameters (0.8 and 3.18 mm). The ^13^N radioactivity intensity (at 3 min intervals) in each ROI tended to increase during the first 21 min of observation (Fig. 2a). Similar trends were revealed by the quantitative analysis of the T65 and C9285 plants. A higher‐to‐lower ^13^N radioactivity intensity gradient from the upper‐to‐lower areas was observed. The ^13^N signal intensity gradually increased over time at each ROI (Fig. 2b,c for C9285; a similar trend was observed for T65, data not shown). Moreover, the diffusion coefficients for the artificial tubes and plants were estimated to compare the gas movement patterns. The diffusion coefficients for the artificial tubes with diameters of 0.8 mm and 3.18 mm were 0.11 ± 0.01 and 0.13 ± 0.004, respectively (Fig. 2d). The diffusion coefficients were also calculated for T65 and C9285 plants in FOV1 (0.15 ± 0.02 and 0.19 ± 0.02, respectively) and FOV2 (0.24 ± 0.09 and 0.12 ± 0.02, respectively) (Fig. 2e,f). There were no significant differences between T65 and C9285 regarding the total amounts of [^13^N]N_2_ gas reaching FOV1 (4.48 ± 0.68 and 6.33 ± 0.39, respectively) within 20 min or FOV2 (1.46 ± 0.07 and 1.67 ± 0.10, respectively) within 40 min after gas feeding (Fig. 3). A series of quantitative analyses indicated the possibility of a similar pattern of [^13^N]N_2_ gas movement among artificial tubes and the T65 and C9285 plants.

### Effects of gas films on [^13^N]N_2_ gas movement

Leaf gas films are layers of air formed on submerged leaves that promote the entry of air into submerged rice plant organs (Raskin & Kende, 1983; Setter *et al*., 1987). To clarify whether the [^13^N]N_2_ signal is derived from the inside of the plant or the gas film on the leaf surface, the gas film was removed from the leaf blade surface of C9285 plants, after which the [^13^N]N_2_ tracer gas was added to the feeding chamber. The removal of the gas film was confirmed by the absence of visual reflections of light on the area treated with Triton X‐100 (Fujifilm Wako Pure Chemical Corp., Tokyo, Japan) just before the PETIS experiment (Fig. 4a). The gas films in submerged plants were removed from 5 cm of the leaf blade from the water surface downward, thereby cutting off the downward [^13^N]N_2_ gas movement from the feeding chamber to the underwater parts through gas films connecting to the air. The [^13^N]N_2_ gas movement was compared between plants with and without intact gas films. The calculated diffusion coefficients were similar between plants with and without gas films at FOV1 (0.08 ± 0.03 and 0.15 ± 0.05, respectively) within 20 min or at FOV2 (0.13 ± 0.002 and 0.11 ± 0.009, respectively) within 40 min (Fig. 4b). There were no significant differences between plants with and without gas films in terms of the ^13^N radioactivity in FOV1 (3.94 ± 1.67 and 4.97 ± 1.90, respectively) and in FOV2 (3.13 ± 1.22 and 2.66 ± 0.89, respectively) (Fig. 4c).

### Comparison of the movement between [^13^N]N_2_ gas and ^11^C photoassimilates

The [^13^N]N_2_ gas movement along the aerenchyma was compared with the ^11^C‐photoassimilate translocation along the phloem in the same test plants (Fig. 5; Video S1). The T65 and C9285 plants presented in Fig. 1(c,d) were fed ^11^CO_2_ tracer gas after the ^13^N radioactivity had decayed sufficiently. The presence of ^11^C photoassimilates in FOV2 was then determined. The ^11^CO_2_ was assimilated immediately after feeding because of the fixation by photosynthesis. The ^11^C photoassimilates were then translocated. Radioactivity was detected earlier after feeding with [^13^N]N_2_ tracer gas than after feeding with ^11^CO_2_ tracer gas (Fig. 5a,b). The [^13^N]N_2_ gas moved and reached the bottom part of the plant through the LS and IN, with marked decreases at the nodes (Fig. 5a, white arrowheads). By contrast, the movement of ^11^C photoassimilates appeared restricted at the nodes, resulting in their accumulation there (Fig. 5b). Moreover, a comparison of the TACs for each ROI of the LS and IN between the ^13^N signals and ^11^C signals confirmed the rapid movement of [^13^N]N_2_ gas (Fig. 6). The ^13^N signals increased rapidly within 20 min after the tracer feeding in the ROIs of the LB and LS in FOV1 (Fig. 6a). The ^13^N signals were detected at *c*. 10 min after the tracer feeding in the LS of FOV2. The ^13^N signals in the ROIs of IN were lower than those in the respective LS, and the arrival time was also delayed (Fig. 6b). Conversely, ^11^C signals were detected *c*. 30 min after the tracer feeding in the ROIs of the LS and IN (Fig. 6c). The [^13^N]N_2_ gas moved through the aerenchyma *c*. three times faster than the ^11^C photoassimilates transported through the phloem. After flushing out of gas, after 20 min of the feeding step, the ^13^N signals decreased in the LB rapidly (Fig. 6a), whereas the ^13^N signals of the LS present in FOV2 continued to increase for more than 10 min before starting to decrease (Fig. 6b). By contrast, the tendency of the ^11^C signals to increase was unaffected by the flushing out of gas (Fig. 6c).

### Gas movement pathways in rice plants

The hollow structures of rice plants developed continuously from the LB to the LS, and the cross‐sectional area of the space gradually increased (Fig. 7a,b). At the junction of the LS and the node, the filling of the parenchyma and the presence of a spongy tissue decreased the space in the aerenchyma (Fig. 7a, bottom), but gas movement was not restricted. The hollow structures connected the LS and the lower IN via a node, and the largest connection areas were positioned near the middle between the two sides of the LS (Fig. 7c). The LS and its inner IN were also connected, and the two hollow structures were joined at the node filled with spongy tissue. The spongy tissue developed in a network toward the middle of the node and served as an intersection for the passage of gas into the aerenchyma (Fig. 7c,d, right). The aerenchyma extended from the LS and IN to the discrimination center and was connected to the root that developed from near the discrimination center node. A substantial abundance of aerenchyma radially connected by parenchyma was detected between the central column and the epidermis of the root (Fig. 7e). With the node as a junction, the aerenchyma in different parts of the rice plant body are connected.

## Discussion

In this study, we used the positron‐emitting [^13^N]N_2_ tracer gas to conduct a PETIS analysis that clarified the gas movement in partially submerged paddy rice T65 and deepwater rice C9285 plants (Fig. 1). Gas diffuses 10^4^‐fold more slowly in water than in air (Armstrong, 1980). Because artificial tubes and plants were submerged in water, the ^13^N signals visualized in the FOVs were assumed to represent [^13^N]N_2_ tracer gas movement through the artificial tubes or the plant aerenchyma. The [^13^N]N_2_ gas is not fixed by rice plants. Elbeltagy *et al*. (2001) estimated the N fixation by inoculated endophytic bacteria *Herbaspirillum* sp. using stable‐isotope ^15^N_2_ in rice plants. The increase in ^15^N concentration was only 0.14% after exposure for 24 h. It is suggested that the effect of ^13^N signals from the N fixation would be less than 1% of the [^13^N]N_2_ gas, and thus it would be hidden by the noise. Therefore, we were able to evaluate the gas movement pattern inside rice plants without any interference from plant biological functions. Additionally, PETIS serial imaging does not require any subsequent processing (e.g. dissection or fixation) after test samples are fed the radiotracer. Hence, the radiotracer observations described herein reflect the aeration of living rice plants. The utility of this system was confirmed by the comparison of the [^13^N]N_2_ gas and ^11^C‐photoassimilate movements in the same plants (Fig. 6). The observations verified the differences in the gas and solid‐state movement patterns in the same test plants. Additionally, the mechanisms underlying the movement of [^13^N]N_2_ and other gases were predicted based on the quantitative results and two‐dimensional segmentation of the X‐ray CT images in this study (Fig. 7).

Because the [^13^N]N_2_ tracer gas fed to control artificial tubes was expected to move via diffusion inside the tubes, the quantitative analysis of ^13^N radioactivity in the tubes reflected the gas diffusion trends (Fig. 2a). The changes in the ^13^N signals in T65 and C9285 plants exhibited a trend similar to that observed in the artificial tubes (Fig. 2b,c). An analysis of the ROIs at the same time points indicated the ^13^N signal intensities decreased from the upper parts to the bottom parts of the artificial tubes and rice plants. The ^13^N signal intensities in the same ROIs gradually increased over time (Fig. 2a–c). The diffusion coefficients calculated for the [^13^N]N_2_ gas movement were of the same order of magnitude in both the artificial tubes and rice plants (Fig. 2d–f). The similarities in the results for the artificial tubes and rice plants suggest that the movement of [^13^N]N_2_ gas in rice plants occurs via diffusion.

However, the mechanisms mediating the movement of other gas species, including nonradioactive N_2_, CO_2_, and O_2_, remain unclear. Regarding O_2_ movement, the concentration of O_2_ is higher in leaves than in other organs, such as the IN and root, because of the O_2_ accumulation resulting from photosynthesis and the uptake from air. Conversely, other organs only consume O_2_ during respiration. These differences can result in higher‐to‐lower O_2_ concentrations from the upper‐to‐lower plant body parts. This O_2_ gradient can induce the diffusion of O_2_. Indeed, the partial O_2_ pressures of paddy rice in air were *c*. 20 kPa in the IN and 11 kPa in the root. These observed differences in O_2_ concentrations may be the driving force for diffusion (Pedersen *et al*., 2009; Mori *et al*., 2019). However, Raskin & Kende (1985) proposed that air is moved by mass flow, rather than by diffusion. They speculated that O_2_ is consumed by respiration and that the subsequently evolved CO_2_ is dissolved in the water surrounding submerged roots. These metabolic processes result in the differences in the volume of air between the upper and lower parts of partially submerged deepwater rice plants. However, the results of the present study provide no evidence for the mass flow of [^13^N]N_2_ gas in partially submerged deepwater rice (Figs 2, 3). We quantitatively analyzed [^13^N]N_2_ gas movement independent of changes in the N_2_ gas composition (Fig. S2). Our results are more supportive that the gas movement occurs via diffusion as a result of partial pressure differences.

The presence of gas films as surface layers in the submerged organs of rice plants plays an important role in enhancement of the O_2_ and CO_2_ supply (Pedersen *et al*., 2009; Mori *et al*., 2019). However, the gas films were retained for 6–8 d during the experimental submergence of the two ecotypes (T65 and C9285), and other genotypes of submerged paddy rice were only retained for 4–7 d in the field (Winkel *et al*., 2014; Mori *et al*., 2019). In the present study, there were no differences in the [^13^N]N_2_ gas movement between with and without an intact gas film (Fig. 4). This suggests that the gas film is more involved in the gas uptake by LBs from surface air layers rather than the passage of gas to the underground plant parts. From the perspective of risk aversion, even if the gas film is eliminated easily, it is clear that the internal hollow structures have an advantage to remain functional in rice plants. On flushing out of gas after 20 min of the tracer feeding, the ^13^N signals at the LB of FOV1 (about 10 cm from the feeding chamber) of the two rice species started to decrease immediately (Fig. 6a). However, the ^13^N signals at the LS of FOV2 (more than 35 cm away from the feeding chamber) took more than 10 min to start to decrease after flushing out (Fig. 6b). The flushing out causes partial pressure differences in the feeding chamber, but it did not alter the tendency of the ^11^C signals to increase. However, as shown in Fig. 6(c), the signal changes of ^11^C within 30 min were not as pronounced as the signal change of [^13^N]N_2_ gas. Therefore, it did not reflect ^11^CO_2_ movement to the underground plant parts, because CO_2_ fixation by photosynthesis was rapid under the light conditions. This indicated that the efficiency of the gas movement of [^13^N]N_2_, O_2_, and CO_2_ is affected by whether these gases are used for plant metabolism. For example, submerged rice took up the O_2_ via ‘snorkeling’ and/or leaf gas film. Deepwater rice adapts to flooding by enhancing rapid elongation of the IN compared with that of paddy rice to allow the ‘snorkeling’ of O_2_ and retaining it in the plant body (Mori *et al*., 2019). Therefore, the metabolic rate and O_2_ partial pressure produced should be different in each part of the submerged tissues. It is suggested that if the present results were replaced with an evaluation of the O_2_ then this may have resulted in an overestimation relative to the O_2_ movement driven by partial pressures generated by plant metabolic activities (e.g. respiration) because there was no [^13^N]N_2_ gas in the plant bodies. Further detailed analyses of O_2_ and CO_2_ metabolic processes and the movement of these gases in plant organs will provide insights into the mechanisms controlling gas movement to adapt to flooding in partially submerged rice plants.

The [^13^N]N_2_ gas and ^11^C‐photoassimilate movement patterns differed in this study (Figs 5, 6). The ^11^C photoassimilates accumulated at the nodes (Fig. 5b), possibly for the redistribution of C from the source organs to the sink organs. Moreover, ^13^N signals were detected earlier than the ^11^C‐photoassimilate signals. The [^13^N]N_2_ gas did not accumulate at the nodes, but it passed through the INs (Figs 5a and 6b). However, the signal of [^13^N]N_2_ gas continued to increase for more than 10 min after the flushing out after 20 min in the LS and IN of FOV2. Although, the effect of flushing out started to appear and the signal started to decrease after 35 min, the decrease of IN was more gradual than in LB and LS. Rice plants have a structural advantage. Specifically, their aerenchyma network facilitates the efficient distribution of gases to the roots and other plant body organs (Fig. 7). The sponge tissue in the node decreases the efficiency of gas movements to the underground plant parts, but it may have a function to minimize gas loss to the atmosphere (Arikado *et al*., 1990). Plants in flood‐prone areas tend to have a constitutive aerenchyma, the formation of which is further induced by the submergence of the roots (Visser *et al*., 2000; Shiono, 2016) and shoots (Parlanti *et al*., 2011; Steffens *et al*., 2011). Therefore, because of the presence of aerenchyma for the diffusion of gases, plants can efficiently take up gaseous substrates (e.g. O_2_ and CO_2_) and maintain their metabolic activities in the underwater tissue.

In the current study, there were no major differences in the [^13^N]N_2_ gas movement between the paddy rice T65 and deepwater rice C9285 (Figs 1, 2, 3). In addition, no significant differences were found between the two varieties in the expression levels of ethylene biosynthesis genes and genes involved in hydrogen peroxide production (Fig. S3). It has been previously reported that there is no difference in ethylene biosynthesis between these two varieties (Hattori *et al*., 2009). Moreover, no significant difference in porosity of these varieties was detected, except for INs (Fig. S4). However, the porosity of LS increased under submergence, and the rates were same in both genotypes. In the PETIS experiment, the IN length was about 1.5 times longer in C9285 than in T65 (Fig. S1). These results suggested that the morphological differences in T65 and C9285 under submergence are limited to the IN and that there is no difference in the rate of gas diffusion between the two genotypes. Therefore, it seems that the INs of deepwater rice function to push leaves above the water surface by elongation and as a simple pathway for gases that diffuse from the leaves rather than to actively transport gases. Although we concluded that the gas diffusion process in submerged plant tissue is not significantly different between the two varieties, the large porosity in the IN such as in deepwater rice may have an advantage to adapt to a flooding environment in terms of being able to retain more gas.

In conclusion, we explored the long‐distance gas movement from the organs above the water to the submerged organs in partially submerged paddy rice and deepwater rice plants. The movement was presumably mediated by diffusion. This is the first study to use [^13^N]N_2_ tracer gas to produce serial PETIS images of gas movement in plants. The efficiency of transporting substrates in a gaseous state was revealed by the faster movement of [^13^N]N_2_ gas than of ^11^C‐photoassimilates inside rice plants. Therefore, plants growing in flood‐prone areas likely evolved a highly efficient systemic aerenchyma network that facilitates O_2_ movement to maintain respiration in submerged organs. These direct observations using live imaging methods provide further insights regarding the thoroughly studied physiological significance of aeration in rice plants. They also represent evidence of the role for aeration in plants that is supported by anatomical studies.

## Author contributions

Y‐GY, YMori, NS, MA, KN and NK conceived and designed the research. Y‐GY, YMori, NS, KK, YMiyoshi, MA, KN and NK conducted the imaging experiments. Y‐GY, YMiyoshi, NS, MY, YN and NK analyzed the image data. Y‐GY, YMori, KN and NK wrote the manuscript, which was reviewed by the other authors. Y‐GY and YMori contributed equally to this work.

## Supporting information


**Fig. S1** Experimental setup of two rice varieties for PETIS imaging.
**Fig. S2** Quantitative analysis of [^13^N]N_2_ diffusion coefficients for different [^13^N]N_2_ tracer gas compositions in the artificial tubes.
**Fig. S3** Quantification of gene expression levels in shallow‐water (SW) and deep‐water (DW) conditions.
**Fig. S4** Porosity at various tissues of shoot in shallow‐water (SW) and deep‐water (DW) conditions.

**Video S1** A typical movie of [^13^N]N_2_ gas or ^11^C‐photoassimilate movement in submerged T65 and C9285 plants.
Please note: Wiley Blackwell are not responsible for the content or functionality of any Supporting Information supplied by the authors. Any queries (other than missing material) should be directed to the *New Phytologist* Central Office.

## References

[nph17726-bib-0001] Arikado H , Ikeda K , Taniyama T . 1990. Anatomico‐ecological studies on the aerenchyma and the ventilating system in rice plants. Bulletin of the Faculty of Bioresources, Mie University 3: 1–24.

[nph17726-bib-0002] Armstrong W . 1980. Aeration in higher plants. Advances in Botanical Research 7: 225–332.

[nph17726-bib-0003] Colmer TD , Pedersen O . 2008a. Underwater photosynthesis and respiration in leaves of submerged wetland plants: gas films improve CO_2_ and O_2_ exchange. New Phytologist 177: 918–926. 18086222 10.1111/j.1469-8137.2007.02318.x

[nph17726-bib-0004] Colmer TD , Pedersen O . 2008b. Oxygen dynamics in submerged rice (*Oryza sativa*). New Phytologist 178: 326–334. 18248586 10.1111/j.1469-8137.2007.02364.x

[nph17726-bib-0005] Elbeltagy A , Nishioka K , Sato T , Suzuki H , Ye B , Hamada T , Isawa T , Mitsui H , Minamisawa K . 2001. Endophytic colonization and in planta nitrogen fixation by a *Herbaspirillum* sp. isolated from wild rice species. Applied and Environmental Microbiology 67: 5285–5293. 11679357 10.1128/AEM.67.11.5285-5293.2001 PMC93302

[nph17726-bib-0006] Fujimaki S , Suzui N , Ishioka NS , Kawachi N , Ito S , Chino M , Nakamura SI . 2010. Tracing cadmium from culture to spikelet: noninvasive imaging and quantitative characterization of absorption, transport, and accumulation of cadmium in an intact rice plant. Plant Physiology 152: 1796–1806. 20172965 10.1104/pp.109.151035 PMC2850040

[nph17726-bib-0007] Hattori Y , Nagai K , Furukawa S , Song X‐J , Kawano R , Sakakibara H , Wu J , Matsumoto T , Yoshimura A , Kitano H *et al*. 2009. The ethylene response factors *SNORKEL1* and *SNORKEL2* allow rice to adapt to deep water. Nature 460: 1026–1030. 19693083 10.1038/nature08258

[nph17726-bib-0008] Hidaka K , Miyoshi Y , Ishii S , Suzui N , Yin Y‐G , Kurita K , Nagao K , Araki T , Yasutake D , Kitano M *et al*. 2019. Dynamic analysis of photosynthate translocation into strawberry fruits using non‐invasive ^11^C‐labeling supported with conventional destructive measurements using ^13^C‐labeling. Frontiers in Plant Science 9: e1946. 10.3389/fpls.2018.01946 PMC6338039 30687351

[nph17726-bib-0009] Kawachi N , Suzui N , Ishii S , Ito S , Ishioka NS , Yamazaki H , Hatano‐Iwasaki A , Ogawa K , Fujimaki S . 2011. Real‐time whole‐plant imaging of ^11^C translocation using positron‐emitting tracer imaging system. Nuclear Instruments and Methods in Physics Research, Section A 648: S317–S320.

[nph17726-bib-0010] Mommer L , Visser EJW . 2005. Underwater photosynthesis in flooded terrestrial plants: a matter of leaf plasticity. Annals of Botany 96: 581–589. 16024559 10.1093/aob/mci212 PMC4247027

[nph17726-bib-0011] Mori Y , Kurokawa Y , Koike M , Malik AI , Colmer TD , Ashikari M , Pedersen O , Nagai K . 2019. Diel O_2_ dynamics in partially and completely submerged deepwater rice: leaf gas films enhance internodal O_2_ status, influence gene expression and accelerate stem elongation for ‘snorkelling’ during submergence. Plant and Cell Physiology 60: 973–985. 30668838 10.1093/pcp/pcz009

[nph17726-bib-0012] Parlanti S , Kudahettige NP , Lombardi L , Mensuali‐Sodi A , Alpi A , Perata P , Pucciariello C . 2011. Distinct mechanisms for aerenchyma formation in leaf sheaths of rice genotypes displaying a quiescence or escape strategy for flooding tolerance. Annals of Botany 107: 1335–1343. 21489969 10.1093/aob/mcr086 PMC3101152

[nph17726-bib-0013] Pedersen O , Rich SM , Colmer TD . 2009. Surviving floods: leaf gas films improve O_2_ and CO_2_ exchange, root aeration, and growth of completely submerged rice. The Plant Journal 58: 147–156. 19077169 10.1111/j.1365-313X.2008.03769.x

[nph17726-bib-0014] Raskin I , Kende H . 1983. How does deep water rice solve its aeration problem. Plant Physiology 72: 447–454. 16663023 10.1104/pp.72.2.447 PMC1066254

[nph17726-bib-0015] Raskin I , Kende H . 1984. Regulation of growth in stem sections of deep‐water rice. Planta 160: 66–72. 24258373 10.1007/BF00392467

[nph17726-bib-0016] Raskin I , Kende H . 1985. Mechanism of aeration in rice. Science 228: 327–329. 17790236 10.1126/science.228.4697.327

[nph17726-bib-0017] Setter TL , Kupkanchanakul T , Kupkanchanakul K , Greenway H . 1987. Concentrations of CO_2_ and O_2_ in floodwater and in internodal lacunae of floating rice growing at 1–2 meter water depths. Plant, Cell & Environment 10: 767–776.

[nph17726-bib-0018] Shiono K . 2016. A barrier to radial oxygen loss enables wetland plants to grow under waterlogged conditions. Root Research 25: 47–62.

[nph17726-bib-0019] Steffens B , Geske T , Sauter M . 2011. Aerenchyma formation in the rice stem and its promotion by H_2_O_2_ . New Phytologist 190: 369–378. 21039565 10.1111/j.1469-8137.2010.03496.x

[nph17726-bib-0020] Thomson CJ , Armstrong W , Waters I , Greenway H . 1990. Aerenchyma formation and associated oxygen movement in seminal and nodal roots of wheat. Plant, Cell & Environment 13: 395–403.

[nph17726-bib-0021] Uchida H , Okamoto T , Ohmura T , Shimizu K , Satoh N , Koike T , Yamashita T . 2004. A compact planar positron imaging system. Nuclear Instruments and Methods in Physics Research Section A: Accelerators, Spectrometers, Detectors and Associated Equipment 516: 564–574.

[nph17726-bib-0022] Visser EJW , Colmer TD , Blom CWPM , Voesenek LACJ . 2000. Changes in growth, porosity, and radial oxygen loss from adventitious roots of selected mono and dicotyledonous wetland species with contrasting types of aerenchyma. Plant, Cell & Environment 23: 1237–1245.

[nph17726-bib-0023] Winkel A , Colmer TD , Ismail AM , Pedersen O . 2013. Internal aeration of paddy field rice (O*ryza sativa*) during complete submergence – importance of light and floodwater O_2_ . New Phytologist 197: 1193–1203. 23215967 10.1111/nph.12048

[nph17726-bib-0024] Winkel A , Pedersen O , Ella E , Ismail AM , Colmer TD . 2014. Gas film retention and underwater photosynthesis during field submergence of four contrasting rice genotypes. Journal of Experimental Botany 65: 3225–3233. 24759881 10.1093/jxb/eru166 PMC4071835

[nph17726-bib-0025] Yamazaki H , Suzui N , Yin Y‐G , Kawachi N , Ishii S , Shimada H , Fujimaki S . 2015. Live‐imaging evaluation of the efficacy of elevated CO_2_ concentration in a closed cultivation system for the improvement of bioproduction in tomato fruits. Plant Biotechnology 32: 31–37.

[nph17726-bib-0026] Yin Y‐G , Ishii S , Suzui N , Igura M , Kurita K , Miyoshi Y , Nagasawa N , Taguchi M , Kawachi N . 2019. On‐line rapid purification of [^13^N]N_2_ gas for visualization of nitrogen fixation and translocation in nodulated soybean. Applied Radiation and Isotopes 151: 7–12. 31151049 10.1016/j.apradiso.2019.05.034

