# Supplementary material for: Noninvasive imaging of hollow structures and gas movement revealed the gas partial‐pressure‐gradient‐driven long‐distance gas movement in the aerenchyma along the leaf blade to submerged organs in rice
Source: New Phytol. 2021 Sep 30;232(5):1974–84. doi: 10.1111/nph.17726 (PMC9293169; doi:10.1111/nph.17726)

New Phytologist

***New Phytologist* Supporting Information**

Article title: **Non-invasive imaging of hollow structures and gas movement revealed the gas partial pressure gradient-driven long-distance gas movement in the aerenchyma along the leaf blade to submerged organs in rice**

Authors: Yong-Gen Yin, Yoshinao Mori, Nobuo Suzui, Keisuke Kurita, MitsutakaYamaguchi, Yuta Miyoshi, Yuto Nagao, Motoyuki Ashikari, Keisuke Nagai, Naoki Kawachi

Article acceptance date: 12 August 2021

The following Supporting Information is available for this article:

Fig. S1 Experimental setup of two rice varieties for PETIS imaging.

Fig. S2 Quantitative analysis of $[^{13}N]N_2$ diffusion coefficients for different $[^{13}N]N_2$ tracer gas compositions in the artificial tubes.

Fig. S3 Quantification of gene expression levels in shallow-water (SW) and deep-water (DW) conditions.

Fig. S4 Porosity at various tissues of shoot in shallow-water (SW) and deep-water (DW) conditions.

Fig. S1 Experimental setup of two rice varieties for PETIS imaging. (a) Paddy rice T65 and (b) deepwater rice C9285. White triangles indicate the node position in each rice plant. There is a leaf sheath above the yellow triangle, but not below it.

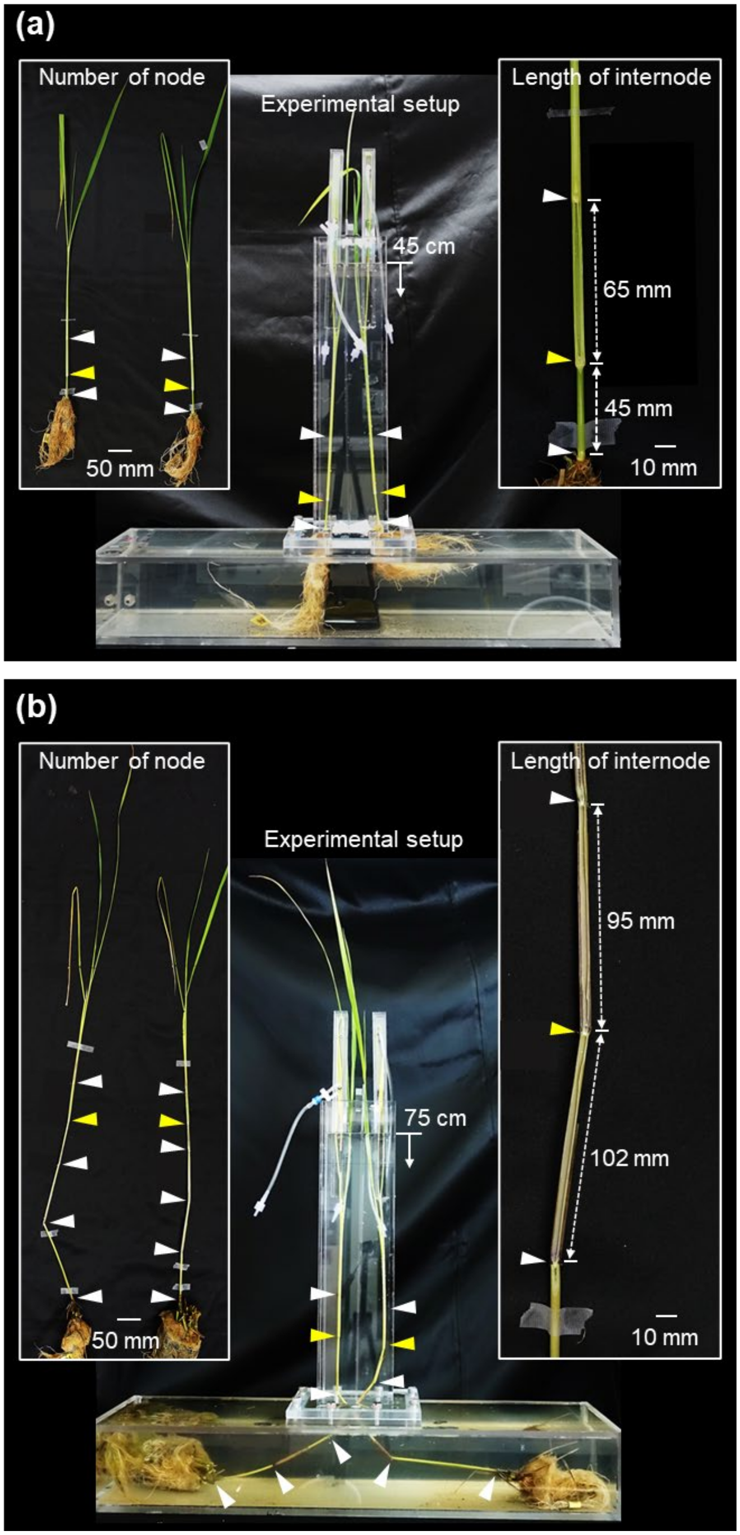

Fig. S2 Several regions of interest (ROIs; yellow square boxes in Fig. 2a) were set for the PETIS images. The ^{13}N radioactivity of each ROI was used to calculate the $[^{13}N]N_2$ gas diffusion coefficient for different N_2 gas compositions (100%, 50%, and 20%) in each artificial tube. Error bars = SE. n = 2.

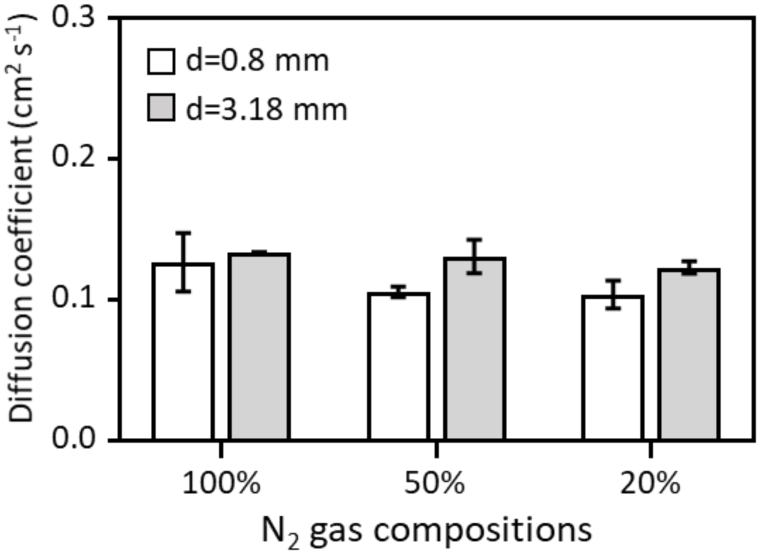

Fig. S3 Quantification of gene expression levels in shallow-water (SW) and deep-water (DW) conditions. a. Expression levels of *ACO7* related to ethylene biosynthesis. b. Expression levels of *OsRBOHB* related to H_2O_2 biosynthesis. Data are mean ± s.d. (n = 3 plants). Total RNA was extracted using a Maxwell RSC Instrument (Promega) according to the manufacturer's protocol. First-strand cDNA was synthesized with the Omniscript RT Kit (Qiagen) and oligo(dT) 20 primers. Quantitative PCR (qPCR) was performed using the StepOne Real-Time PCR System (Applied Biosystems) with Thunderbird SYBR qPCR Mix (Toyobo). Expression levels were normalized to that of RICE UBIQUITIN 2. PCR was performed using specific primers for *ACO7* (ACO7-F; GGAGATTCCAGTGATTGATC and ACO7-R; GGTTTTCCACCCAGAAGAAG), *OsRBOHB* (RBOHB-F; ATGGCTGACCTGGAAGCAG and RBOHB-R; AGTGTGGCTGCGTCATCTTG) and RICE *UBIQUITIN 2* (Ubi-F; GAGCCTCTGTTCGTCAAGTA and Ubi-R; ACTCGATGGTCCATTAAACC).

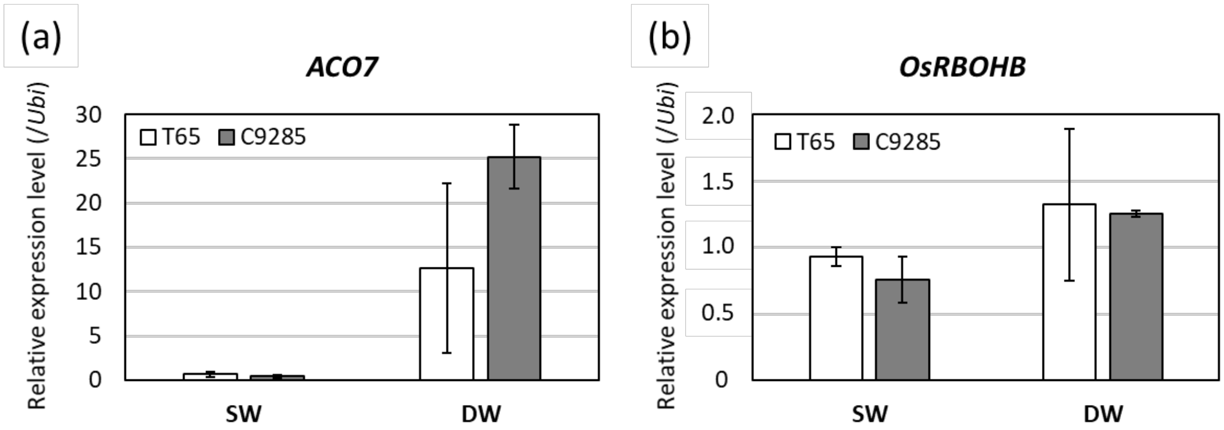

Fig. S4 Porosity at various tissues of shoot in shallow-water (SW) and deep-water (DW) conditions. One-month-old plants were grown in shallow water (5 cm water level from the soil surface) or deep water (80% submergence of plants) for one month. The middle bar represents the mean and the error bars represent s.d. (n=3 plants). Porosity (%) gas spaces per unit tissue volume) was measured for leaf sheath, leaf blade and internode, by determining plant tissue buoyancy before and after vacuum infiltration of the gas spaces with water (Visser and Bogemann, 2003). Triton X at 0.05% was used to remove surface gas films on leaf segments (Winkel et al., 2011). Leaf sheath and leaf blade were cut into 50 mm segments for the measurements; only mid-leaf segments were used for leaf porosity measurements. The porosity of the internodes was investigated by cutting out one internode that contained nodes above and below it. One-way analysis of variance (ANOVA) followed by Tukey's multiple-comparison test. Asterisk (*) indicates significant differences ($P < 0.05$).

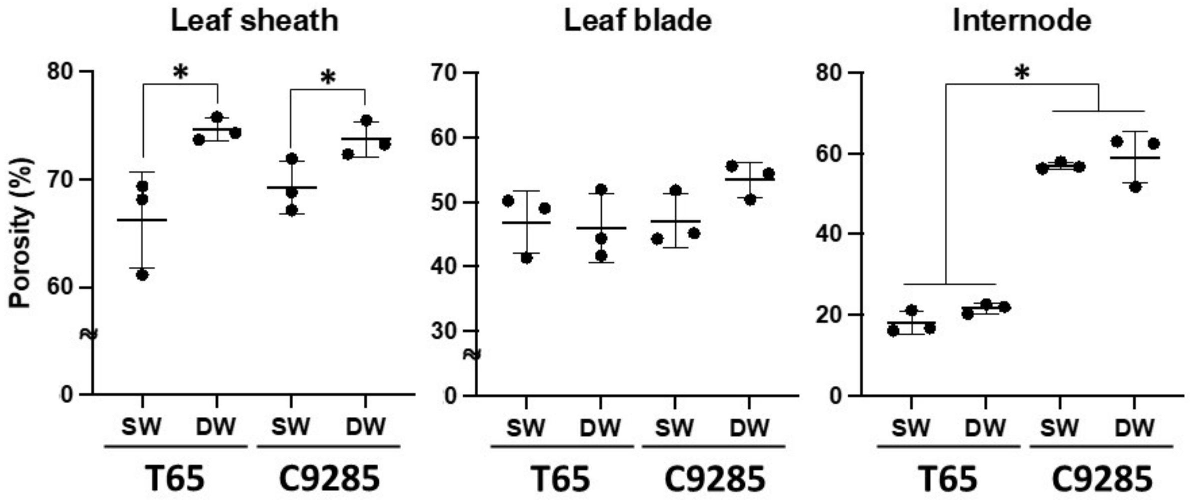

Supplement: Supplementary file 1 — Fig. S1 Experimental setup of two rice varieties for PETIS imaging. Fig. S2 Quantitative analysis of [13N]N2 diffusion coefficients for different [13N]N2 tracer gas compositions in the artificial tubes. Fig. S3 Quantification of gene expression levels in shallow‐water (SW) and deep‐water (DW) conditions. Fig. S4 Porosity at various tissues of shoot in shallow‐water (SW) and deep‐water (DW) conditions. [file NPH-232-1974-s002.pdf]
